# Construction of an endoplasmic reticulum stress-related signature in lung adenocarcinoma by comprehensive bioinformatics analysis

**DOI:** 10.1186/s12890-023-02443-2

**Published:** 2023-05-15

**Authors:** Yang Wang, Jun Nie, Ling Dai, Weiheng Hu, Sen Han, Jie Zhang, Xiaoling Chen, Xiangjuan Ma, Guangming Tian, Di Wu, Ziran Zhang, Jieran Long, Jian Fang

**Affiliations:** 1 Key Laboratory of Carcinogenesis and Translational Research (Ministry of Education/Beijing), Department of Thoracic Oncology, Peking University Cancer Hospital & Institute, 52# Fucheng Road, Haidian District, Beijing, 100142 China; 2 Clinical Trial Center, Peking University Cancer Hospital & Institute, Beijing, China

**Keywords:** Lung adenocarcinoma, Endoplasmic reticulum stress, Risk model, Prognosis, Bioinformatics

## Abstract

**Background:**

Lung Adenocarcinoma (LUAD) is a major component of lung cancer. Endoplasmic reticulum stress (ERS) has emerged as a new target for some tumor treatments.

**Methods:**

The expression and clinical data of LUAD samples were downloaded from The Cancer Genome Atlas (TCGA) and The Gene Expression Omnibus (GEO) database, followed by acquiring ERS-related genes (ERSGs) from the GeneCards database. Differentially expressed endoplasmic reticulum stress-related genes (DE-ERSGs) were screened and used to construct a risk model by Cox regression analysis. Kaplan–Meier (K-M) curves and receiver operating characteristic (ROC) curves were plotted to determine the risk validity of the model. Moreover, enrichment analysis of differentially expressed genes (DEGs) between the high- and low- risk groups was conducted to investigate the functions related to the risk model. Furthermore, the differences in ERS status, vascular-related genes, tumor mutation burden (TMB), immunotherapy response, chemotherapy drug sensitivity and other indicators between the high- and low- risk groups were studied. Finally, quantitative real-time polymerase chain reaction (qRT-PCR) was used to validate the mRNA expression levels of prognostic model genes.

**Results:**

A total of 81 DE-ERSGs were identified in the TCGA-LUAD dataset, and a risk model, including HSPD1, PCSK9, GRIA1, MAOB, COL1A1, and CAV1, was constructed by Cox regression analysis. K-M and ROC analyses showed that the high-risk group had a low survival, and the Area Under Curve (AUC) of ROC curves of 1-, 3- and 5-years overall survival was all greater than 0.6. In addition, functional enrichment analysis suggested that the risk model was related to collagen and extracellular matrix. Furthermore, differential analysis showed vascular-related genes FLT1, TMB, neoantigen, PD-L1 protein (CD274), Tumor Immune Dysfunction and Exclusion (TIDE), and T cell exclusion score were significantly different between the high- and low-risk groups. Finally, qRT-PCR results showed that the mRNA expression levels of 6 prognostic genes were consistent with the analysis.

**Conclusion:**

A novel ERS-related risk model, including HSPD1, PCSK9, GRIA1, MAOB, COL1A1, and CAV1, was developed and validated, which provided a theoretical basis and reference value for ERS-related fields in the study and treatment of LUAD.

**Supplementary Information:**

The online version contains supplementary material available at 10.1186/s12890-023-02443-2.

## Introduction

Lung cancer is one of the most common malignant cancers and is the leading cause of cancer-related death. In United States, it is estimated that 350 people will die from lung cancer every day in 2022 [1]. In China, the incidence and mortality of lung cancer was highest among all kinds of malignant tumors [2]. As the major pathological type of lung cancer, the incidence of lung adenocarcinoma (LUAD) is increasing year by year, accounting for 40% of all lung cancer [3–5]. With the development of target therapy and immunotherapy, the median survival time of advanced LUAD has been prolonged, but unfortunately, more than half of LUAD can not benefit from these novel therapies. Therefore, we need new methods and tools to predict the survival of LUAD and provide new perspectives for the treatment of LUAD.

Endoplasmic reticulum (ER) is an important organelle in eukaryotic cells, responsible for protein folding, maintaining Ca + homeostasis, and providing a suitable environment for lipid and cholesterol synthesis [6, 7]. Various stimulus signals, such as accumulation of misfolded or unfolded proteins, nutritional deficiencies or hypoxia can put the ER in a stressful state, which is called endoplasmic reticulum stress (ERS) [8]. ERS is involved in the origin and metastasis of cancers including lung, breast, and prostate cancers [9]. ERS-related genes (ERSGs) such as IRE1α could modulate the chemokines related to angiogenesis and pro-inflammatory response and furtherly promote the angiogenesis of glioma [10, 11]. High levels of XBP1 in tumor-infiltrating dendritic cells can boost tumor growth and invasion by suppressing tumor immunity [12]. In addition, severe ERS can also induce immune-related death of tumor cell [13] Importantly, IFN-γ could induce cell apoptosis in LUAD through ERS triggering, and ERSGs have become a potential target for tumor therapy [14]. However, the action mechanism of ERS in LUAD has not been fully elucidated.

To investigate the effect of ERSGs on the prognosis of LUAD patients, we screened differentially expressed endoplasmic reticulum stress-related genes (DE-ERSGs) and constructed a risk model using the LUAD expression data from The Cancer Genome Atlas (TCGA) database. The risk model was validated in the TCGA test set and GSE31210 validation set. In addition, the mRNA expression levels of 6 prognostic genes were verified by quantitative real-time polymerase chain reaction (qRT-PCR). In conclusion, this study provided theoretical basis and reference value for the research and treatment of LUAD.

## Materials and methods

### Data source

The data for this study were obtained from TCGA database (https://portal.gdc.cancer.gov/) and Gene Expression Omnibus (GEO) database (https://www.ncbi.nlm.nih.gov/gds). The TCGA-LUAD dataset includes 59 normal samples and 514 LUAD samples, of which 500 LUAD samples had available survival information. In addition, the GSE31210 dataset, which contains 226 LUAD samples with survival information and clinical data, was used as an external validation set.

### Identification of differentially expressed endoplasmic reticulum stress-related genes (ERS-DEGs)

Differential analysis was performed for normal samples and LUAD patients in the TCGA-LUAD dataset using the “limma” R package with differential screening conditions of* P* < 0.05 and |log_2_FC|> 1. The Heat map and volcano map of differentially expressed genes (DEGs) were plotted by the “ggplot2” (version 3.3.5) R package and the “heatmap” (version 1.0.12) R package [15]. Then we searched for “ER stress” using the GeneCards database (https://www.genecards.org/) and selected genes with a correlation score ≥ 5 to obtain endoplasmic reticulum stress-related genes (ERSGs). To screen out ERS-DEGs, the DEGs were intersected with ERSGs and visualized with a Venn diagram.

### Construction and validation of a risk model

The 500 LUAD samples with survival information in the TCGA-LUAD dataset were randomly divided into a training set and a test set in the ratio of 7:3 (350:150), and 350 samples were used as the training set for univariate cox regression analysis base on ERS-DEGs. The genes with *P* < 0.05 in the univariate Cox regression analysis were subsequently included in the multivariate Cox analysis, using the stepwise regression function STEP with parameter DIREction set to 'BOTH' to adjust the multivariate regression model. The regression coefficients of the prognosis-related genes were combined with the expression levels of the corresponding genes with the following equation to calculate the risk score for each sample.

$$risk score={\sum }_{n=1}^{n}({coef}_{i}\times {x}_{i})$$

Coefficient is the regression coefficient of the i-th gene, x is the expression value of the i-th gene, and n is the number of prognostic genes. Patients were allocated into high-risk and low-risk groups according to the median of the risk scores. To assess the accuracy of the risk model, risk curve was plotted based on risk scores, Kaplan–Meier (K-M) survival analysis was performed to estimate survival differences between high- and low- risk groups, and receiver operating characteristic (ROC) curve was plotted using the “survivvalroc” R package. In addition, t-test was used to estimate correlations between risk model and clinical traits, with *P* < 0.05 represented a significant difference in risk between groups.

To test the applicability of the model, the remaining 150 samples from the TCGA-LUAD dataset were used as a test set and 226 samples from the GSE31210 dataset were used as an independent external validation set. In the test set and validation set, patients were divided into high-risk and low-risk groups based on the median of the calculated risk values respectively, then risk curves were plotted and survival analysis was performed, and ROC curves were plotted. Finally, the relationships between risk scores and clinical characteristics were investigated by the “ggplot2” R package.

### Correlation of risk scores with clinicopathological traits

The clinical traits in the TCGA training set were collated, including, age, sex, disease stage, T, N, and M stage traits. The TCGA training set was grouped by different clinical traits, and the Wilcoxon test was used to compare whether there was a significant difference in the risk values among the different groups.

### Independent prognostic analysis of the risk model

Univariate Cox analysis and multivariate Cox analysis were used to explore the independent prognosis of the risk model and prognostic genes. Clinical traits such as age, gender, STAGE, M, T, N, and riskscore from the TCGA training set were included in the univariate Cox analysis, and subsequently, factors with *P* < 0.05 were included in the multivariate Cox analysis using the stepwise regression function STEP, with the parameter DIREction set to ‘both’. The factors obtained by multivariate cox regression were included in the plotting of a nomogram graph. In the nomogram graph, each factor corresponded to a score, and the total score of each factor was added to the total score, and the 1-year, 3-year, 5-year OS was predicted according to the total score, and the higher the score, the lower the survival rate. Based on the prediction model, calibration curve was plotted to assess the predictive efficiency.

### Enrichment analysis of high- and low- risk groups

The TCGA training set was divided into high- and low- risk groups according to median risk values, 175 in the high-risk group and 175 in the low-risk group, and the “limma” R package was used for differential analysis. To further explore the differences between high- and low- risk groups, we enriched the obtained differential genes (*P* < 0.05 and |log_2_FC|> 1) for high- and low- risk. In this study, we used the online enrichment method gprofiler (https://biit.cs.ut.ee/gprofiler/gost) to find the common functions and related pathways of a large number of genes in the DEGscollection, which contained some common enrichment databases, such as Kyoto Encyclopedia of Genes and Genomes (KEGG) [16–18], Gene Ontology (GO), REACTOME (https://reactome.org), etc. We did GO function annotation and REACTOME analysis for DEGs, and extracted the biological significance of each gene and plotted the bubble map separately.

### Comparison of endoplasmic reticulum stress (ERS) status and vascular-related genes

The expression levels of EIF2AK3, DDIT3, TRIB3, ERN1, ATF4, ATF6, HSPA5, and XBP1 genes are often used as indicators of the intensity of ERS in cells or tissues [19], and the expression levels of these genes in the TCGA training set were extracted. The Wilcoxon test was used to compare the high- and low differences of these genes in the risk groups, visualized as box plots. ERS-induced signal transduction and regulation can promote angiogenesis. Therefore, we also used the Wilcox test to analyze the expression levels of angiogenic factor (VEGF)-related genes: VEGFA, VEGFB, VEGFR1 (FLT1), VEGFR2 (KDR), VEGFR3 (FLT4) [19] and vascular concentration-related genes: CD31 (PECAM1), VWF (CLDN5) [20] between the high- and low- risk groups.

### Comparison of tumor mutation burden (TMB) between high- and low- risk groups

TMB is the number of somatic mutations in the tumor genome after removal of germline mutations [21]. To investigate the TMB situation between high- and low- risk groups, the Wilcoxon test was applied to compare the TMB values of high- and low- risk groups in the TCGA training set. In addition, tumor genomic mutations can cause tumors to express tumor-specific mutant proteins that are not expressed on normal cells, and such proteins are called neoantigens [22]. These neoantigens became compelling immune targets and were obtained from the TCGA database and compared between high- and low- risk groups in the TCGA training set.

### Comparison of immunotherapy response between high- and low- risk groups

Immunotherapy is a treatment method that artificially enhances or suppresses the immune function of the body for the purpose of treating the disease [23]. The target of immunotherapy is the immune system of human bodies. Therefore, reactivating immune cells and reversing the immunosuppressive state of the tumor microenvironment are the important goals of immunotherapy [23]. We used Tumor Immune Dysfunction and Exclusion (TIDE, http://tide.dfci.harvard.edu/) to predict the likelihood of response to immunotherapy. The TIDE scores, the distributions of PD-L1 protein (CD274), T cell dysfunction score and T cell exclusion score for each sample in the TCGA training set can be acquired from The TIDE website [24], and the Wilcoxon test and visualization of these four indicators were carried out.

### Chemotherapy drug susceptibility prediction

The Genomics of Drug Sensitivity in Cancer (GDSC) database contains a large number of drug sensitivity and genomic datasets that are important for the discovery of potential tumor therapeutic targets cells [25]. The database contains information on oncogenomic mutations including oncogenic point mutations, gene amplification and loss, tissue type, and expression profiles. IC50, the half-inhibitory concentration, is the half-inhibitory concentration of the antagonist being measured [26]. We used “pRRopheticPredict” R package (version 0.5) to calculate the differences of 138 drug IC50 in the high- and low- risk groups.

### Quantitative real-time polymerase chain reaction (qRT-PCR) validation

Normal cell lines HBE135-E6E7 as well as LUAD cell lines A549, NCI-H1975, and NCI-1395 were used for PCR to verify the expression of prognostic model genes. Total RNA was extracted from all cell lines with Trizol reagent (CAT.-G356281) provided by ambion company. Then, used sweScript RT I First strabd cDNA SynthesisAll-in-OneTM First-Strand cDNA Synthesis Kit (CAT.-G33330-50) provided by the Servicebio company for reverse transcription reaction. PCR was performed using the 2xUniversal Blue SYBR Green qPCR Master Mix (CAT.-G3326-05) kit provided by Servicebio. The PCR conditions were: 95 ℃ pre-denaturation for 1 min, and then 40 cycles. Each cycle included 95 ℃ denaturation for 20 s, 55 ℃ annealing for 20 s, and 72 ℃ extension for 30 s. GAPDH was used as an internal reference for gene detection. Primer sequences were shown in Supplemental Table 1. Three biological replicates were done in this study. HBE135-E6E7 and the expression of the biomarkers in A549, NCI-H1975, and NCI-1395 cell lines were compared by Analysis of Variance (ANOVA). *P* < 0.05 was considered a significant difference.

**Table 1:** The correlation between the risk score and clinical traits in the GSE31210 dataset

	**Expression**			
	**Total**	**high**	**low**	***P* value**
	**(*N* = 226)**	**(*N* = 113)**	**(*N* = 113)**	
**Gender**				
Female	121 (53.5%)	50 (44.2%)	71 (62.8%)	0.00764
Male	105 (46.5%)	63 (55.8%)	42 (37.2%)	
**age (years)**				
> = 60	130 (57.5%)	67 (59.3%)	63 (55.8%)	0.686
< 60	96 (42.5%)	46 (40.7%)	50 (44.2%)	
**Stage**				
I	168 (74.3%)	69 (61.1%)	99 (87.6%)	< 0.001
II	58 (25.7%)	44 (38.9%)	14 (12.4%)	

### Statistical analysis

All open databases and R software were utilized to analyze and visualize in this study. T-test was utilized to evaluate the relevance of risk model and clinical traits. The discrepancies of genes’ expression between high- and low-risk groups were mined via Wilcoxon test. If not specified above, a p-value less than 0.05 was considered statistically significant.

## Results

### Identification of ERS-DEGs

**Fig. 1:**
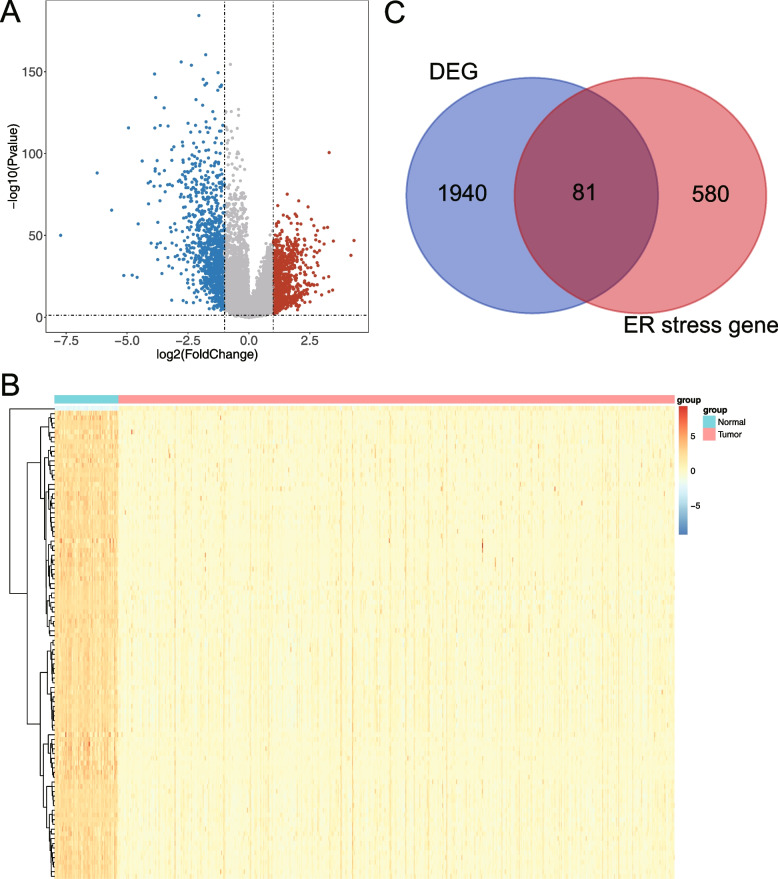
Identification of ERS-DEGs. **A** The volcano map of DEGs between normal and LUAD patients. **B** The heat map of DEGs between normal and LUAD patients. **C** The Venn diagram of ERSGs and DEGs

A total of 2021 DEGs were counted between normal and LUAD samples, with 918 upregulated genes and 1103 downregulated genes. The volcano and heat maps showed the expression of top100 DEGs between normal and LUAD patients (Fig. 1A and B). 661 ERSGs with a correlation score ≥ 5 in the GeneCards database. Next, we took the intersection of the 661 ERSGs and the set of 2021 DEGs to obtain 81 ERS-DEGs for downstream analysis, and the Venn diagram was shown in Fig. 1C.

### Construction and validation of a risk model

**Fig. 2:**
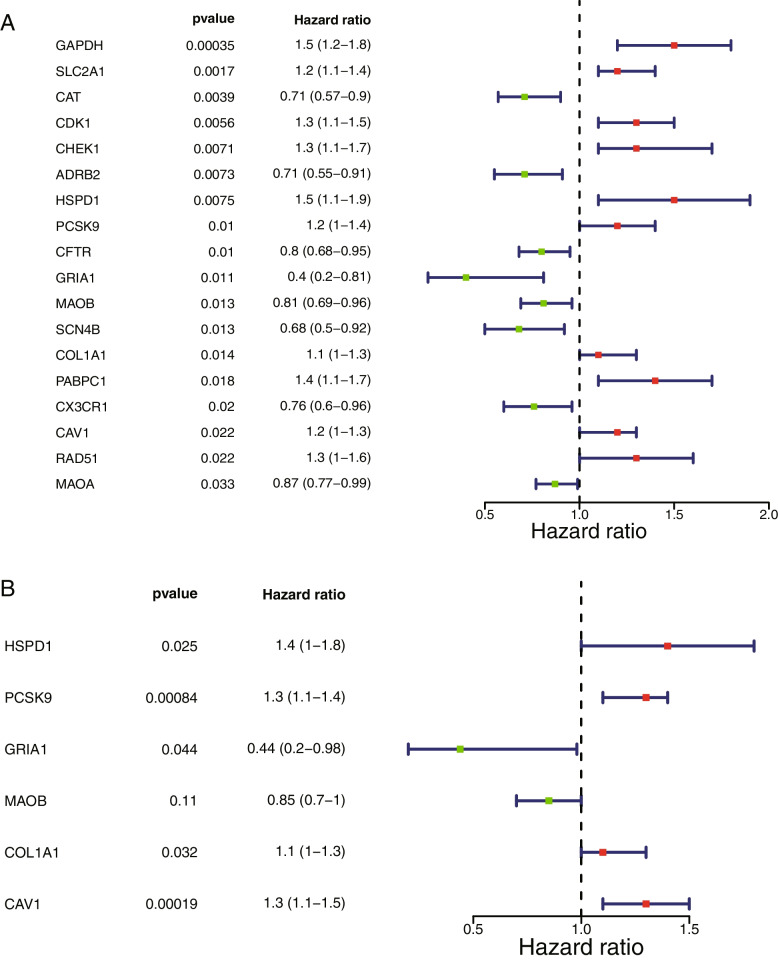
Identification of model genes by univariate Cox regression and multivariate Cox analysis. **A** The forest map of univariate Cox analysis. **B** The forest map of multivariate Cox analysis

**Fig. 3:**
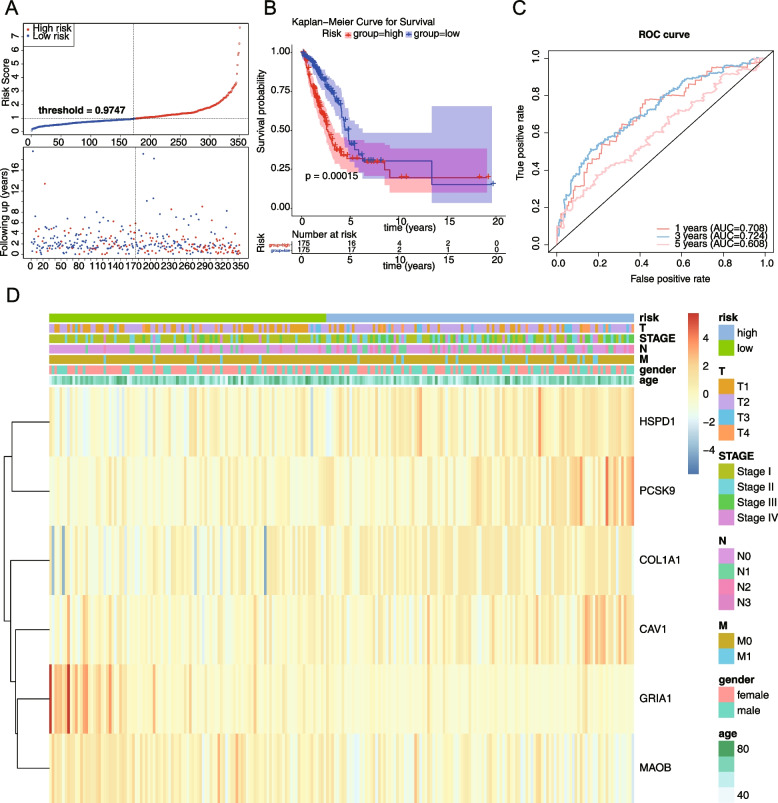
The validation of the risk model in training set. survival differences in high-risk and low-risk groups of training set. **A** The risk profile of training set. **B** The KM survival curve in high-risk and low-risk groups of training set. **C** The ROC curve of training set. **D** Heat map of correlation between iskscore and each clinical characteristic of training set

**Fig. 4:**
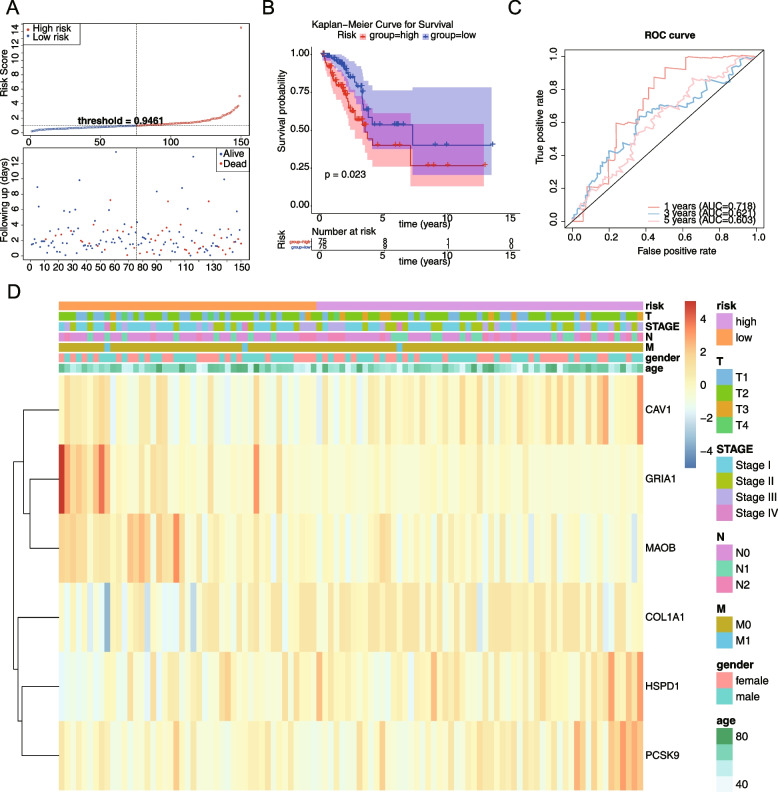
The survival differences in high-risk and low-risk groups of test set. **A** Kaplan–Meier Curve for Survival. **B** Risk Curve. **C** Correlation heatmap of different modules and clinical parameters. **D** the ROC curve

**Fig. 5:**
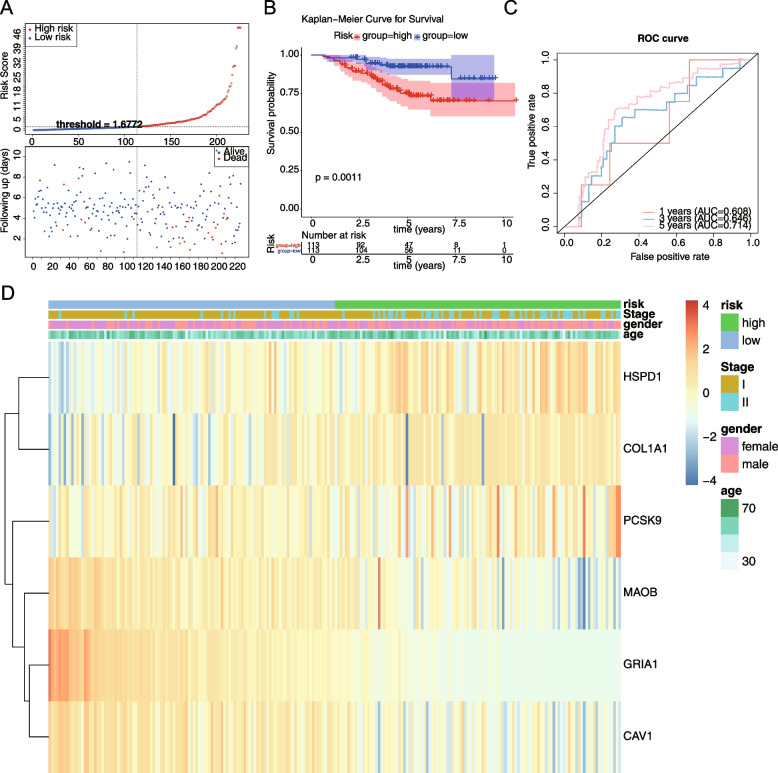
The survival differences in high-risk and low-risk groups of external validation set. **A** Kaplan–Meier Curve for Survival. **B** Risk Curve. **C** Correlation heatmap of different modules and clinical parameters. **D** the ROC curve

A total of 18 genes were screened by univariate Cox regression, and the results were shown in Fig. 2A (Supplemental Table 2). A total of 6 genes appeared in the results of multivariate cox analysis: HSPD1, PCSK9, GRIA1, MAOB, COL1A1, and CAV1. These genes were used as prognostic factors in this study to construct the risk model, and the results were shown in Fig. 2B (Supplemental Table 3). Based on the expression of the model genes and the regression coefficients obtained from multivariate cox regression, the following risk score formula was obtained risk score = [0.3207 × mRNA expression level of HSPD1] + [0.124 × mRNA expression level of COL1A1] + [0.233 × mRNA expression level of PCSK9] + [-0.159 × mRNA expression level of MAOB] + [-0.822 × mRNA expression level of GRIA1] + [0.251 × mRNA expression level of CAV1]. Using the median risk score as the optimal threshold, patients were divided into high-risk and low-risk groups, and the risk profile (Fig. 3A) and KM survival curve (Fig. 3B) demonstrated that there was a remarkable difference in patient survival between the high-risk groups (*P* < 0.05), and patients in the high-risk group had a lower overall survival. The ROC curve had an Area Under Curve (AUC) greater than 0.6 at 1, 3, and 5 years (Fig. 3C), and the t-test was used to assess the risk model was correlated with clinical traits, and as seen in the table, there were significant discrepancies in N-stage and stage staging between the high- and low- risk groups (Fig. 3D, Supplemental Table 4). In the TCGA test set, risk curves and KM survival curves demonstrated significant discrepancies between high- and low risk groups, with AUCs greater than 0.6 at 1, 3, and 5 years, indicating that the risk model constructed can be effectively used as a prognostic model (Fig. 4A-D). And in the GSE31210 dataset, the overall survival was lower in the high-risk group compared with the low-risk group. the AUC at 1, 3, and 5 years were 0.608, 0.646, and 0.714, respectively. The results of the correlation between the risk model and clinical traits showed that there was a remarkable difference between the high- and low-risk groups in STAGE (Fig. 5A-D, Table 1).

### Correlation of risk scores with clinicopathological traits

**Fig. 6:**
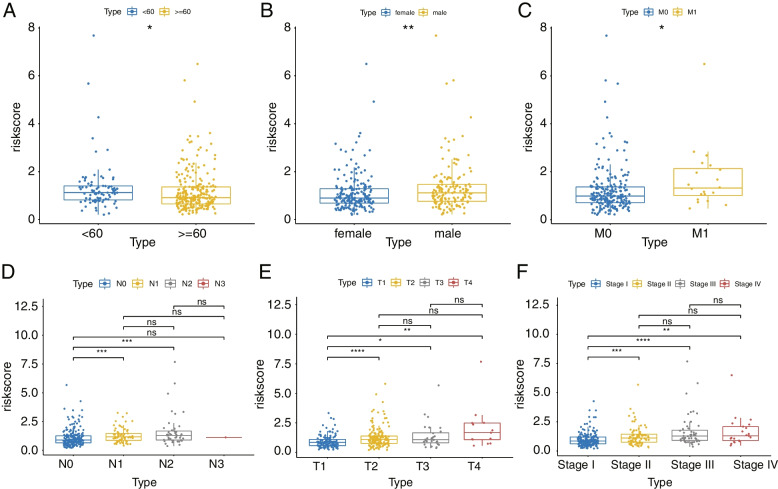
The box plots of risk scores in **A** Age. **B** gender. **C** M stage. **D** N stage. **E** T stage. **F** tumor stages

The results of the correlation between risk model and clinical traits showed significant differences in risk values between the age < 60 and >  = 60 groups, between gender groups, among Stage I-Stage II, Stage II-Stage III, Stage I-Stage III, between the M0, M1 subperiods, among the N0-N1 and N0-N2 subperiods, and between the T1-T2, T1-T3, and T1-T4 subperiods (Fig. 6A-F). Therefore, the risk model correlates with age, sex, disease stage, T stage, N stage, and M stage.

### Independent prognostic analysis of the risk model

**Fig. 7:**
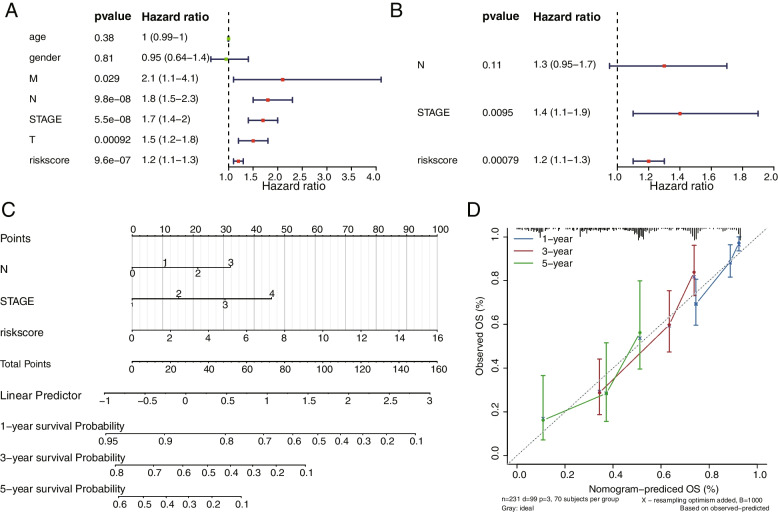
Independent prognostic analysis of the risk model in validation set. **A** The forest plot of Univariate Cox analysis. **B** The forest plot of Multivariate Cox analysis. **C** Nomogram graph predicting survival. **D** Calibration curve for the nomogram

The results of the univariate Cox analysis showed that the clinical traits stage, M, T, N, and risk score were significant (Fig. 7A, Supplemental Table 5). Adjusting for the multivariate regression model, stage, N, and risk score values appeared in the results of the multivariate Cox analysis (Fig. 7B, Supplemental Table 6). The nomogram graph were shown in Fig. 7C. In the corrected curve, the c-index of the model was 0.724 and the corrected c-index was 0.717, and the slopes were calculated to be 0.693, 0.293, and 0.169 at 1, 3, and 5 years, respectively, indicating that the best prediction was achieved at 1 year (Fig. 7D).

### Enrichment analysis of high- and low- risk groups

**Fig. 8:**
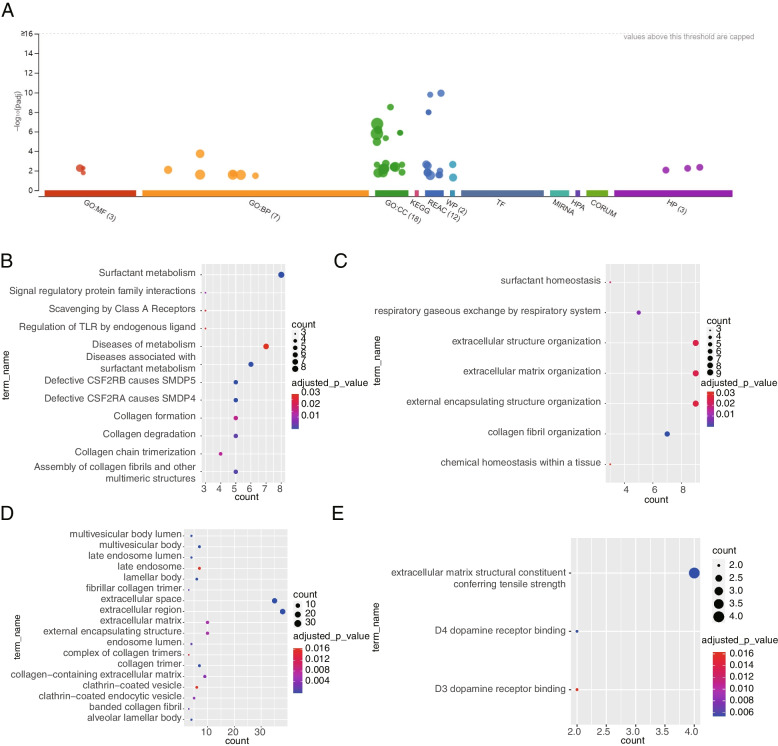
The enrichment Analysis of 70 DEGs between high and low risk groups. **A** Overview of enrichment results. **B** The bubble diagram of GO-BP enriched by DEGs. **C** The bubble diagram of GO-CC enriched by DEGs. **D** The bubble diagram of GO-MF enriched by DEGs **E** The bubble diagram of REACTOME enriched by DEGs

The TCGA training set was divided into a high-risk group containing 175 samples and a low-risk group containing 175 samples according to the median risk value, and the differential analysis yielded a total of 70 difference genes. The enrichment analysis of the above obtained 70 differential genes resulted in 45 relevant entries (Fig. 8A). The results of REACTOME demonstrated that 70 differential genes were correlated with pathways related to surfactant and collagen, such as surfactant metabolism, diseases associated with surfactant metabolism, collagen formation, and collagen degradation and so on (Fig. 8B). GO results revealed that these genes were associated with extracellular organization of structure and matrix collagen fibril organization, and chemical homeostasis within a tissue in biological process (BP) terms (Fig. 8C). As for cellular components (CC), differential genes were associated with body lumen, endosome, and extracellular matrix-related pathways (Fig. 8D). Meanwhile, extracellular matrix structural constituent conferring tensile strength and D3/D4 dopamine receptor binding were enriched in these genes for molecular functions (MF) terms (Fig. 8E). In summary, the above mentioned functions and pathways were associated with the occurrence and development of LUAD.

### Comparison of ERS and vascular-related genes

**Fig. 9:**
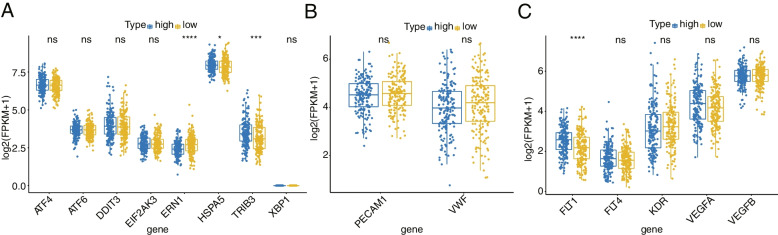
Comparison of ERS and vascular-related genes. **A** The box line plot of genes related to endoplasmic reticulum stress state in high and low risk groups. **B** The box plot of vascular-related genes in high and low risk groups

As shown in the Fig. 9A, three of the ERS-related genes were significantly different between the high- and low- risk groups, namely ERN1, TRIB3, and HSPA5 genes. In the vascular-related genes, FLT1 was significantly different between the high- and low- risk groups (Fig. 9B).

### Comparison of TMB and immunotherapy response between high- and low risk groups

**Fig. 10:**
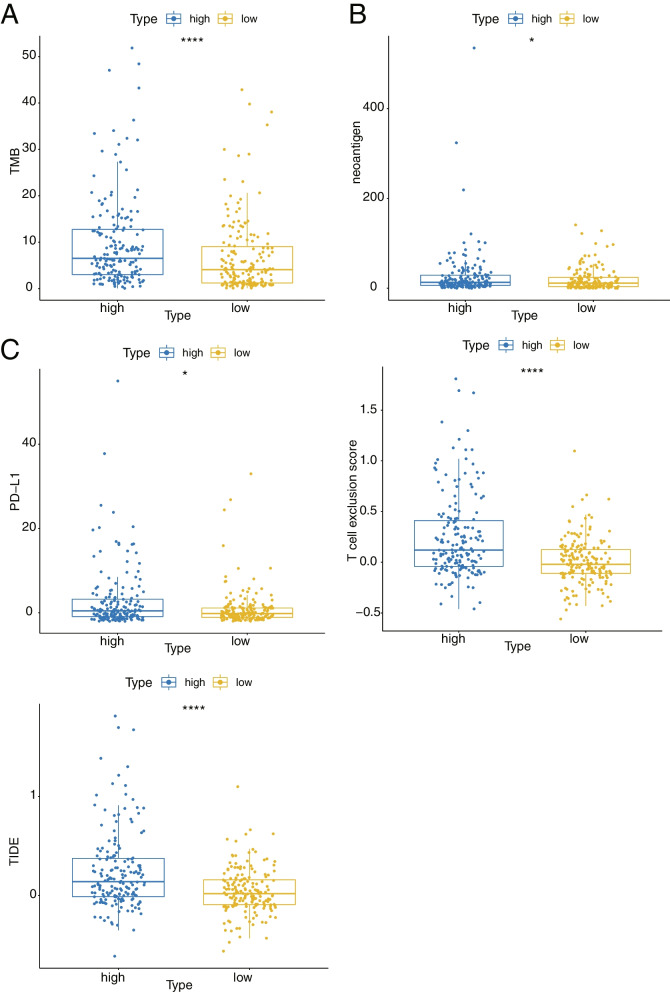
Comparison of TMB and immunotherapy response between high and low risk groups. **A** The Box plot of TMB values in the high and low risk groups. **B** The Box plot of neoantigen in the high and low risk groups. **C** The Box plot of TIDE score, PD-L1 protein (CD274), and T cell exclusion score in the high and low risk groups

TMB is a quantitative biomarker that reflects the total number of mutations carried by tumor cells. Tumor cells with high- TMB have higher levels of neoantigens, which help the immune system recognize tumors and stimulate proliferation of anti-tumor T cells and anti-tumor responses. The results of the TMB in the high- and low- risk groups were shown in the Fig. 10A, indicating a significant discrepancies in TMB between the high- and low- risk groups, and the neoantigen comparison also indicated a significant difference in neoantigen between the high- and low- risk groups (Fig. 10B). TIDE was used to predict the likelihood of response to immunotherapy. As seen in the Fig. 10C, there was a notable difference in the TIDE score and PD-L1 protein (CD274), and T cell exclusion score between the high- and low- risk groups.

### Chemotherapy drug susceptibility prediction

**Fig. 11:**
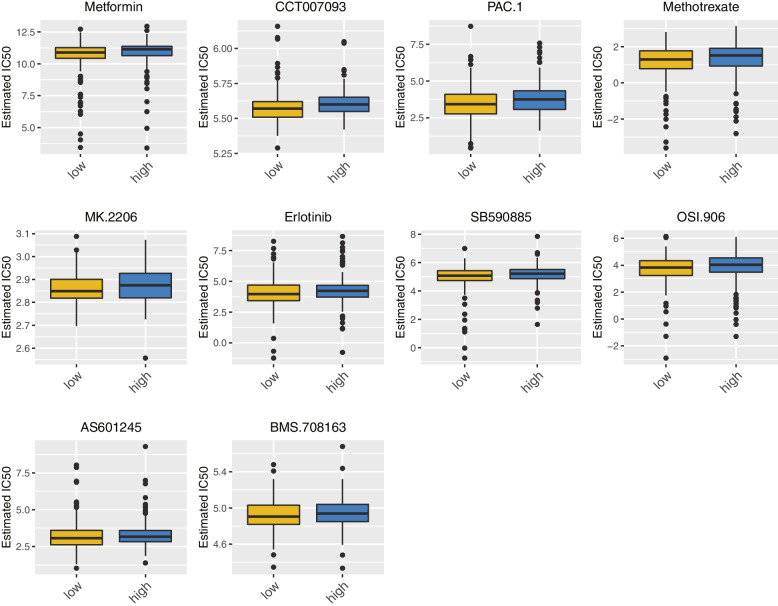
The box plot of the 10 drugs with significant differences in the high and low risk groups

The GDSC data came from 75,000 trials describing the reactions of about 200 anticancer drugs in more than 1,000 tumor cells. The IC50 of the drugs in different groups was calculated to obtain the significant difference between high- and low risk groups. The results showed that there were 10 drugs with significant discrepancies in the high- and low- risk groups, namely Metformin, CCT007093, PAC.1, Methotrexate, MK.2206, Erlotinib, SB590885, OSI.906, AS601245, and BMS.708163 (Fig. 11).

### qPCR validation

**Fig. 12:**
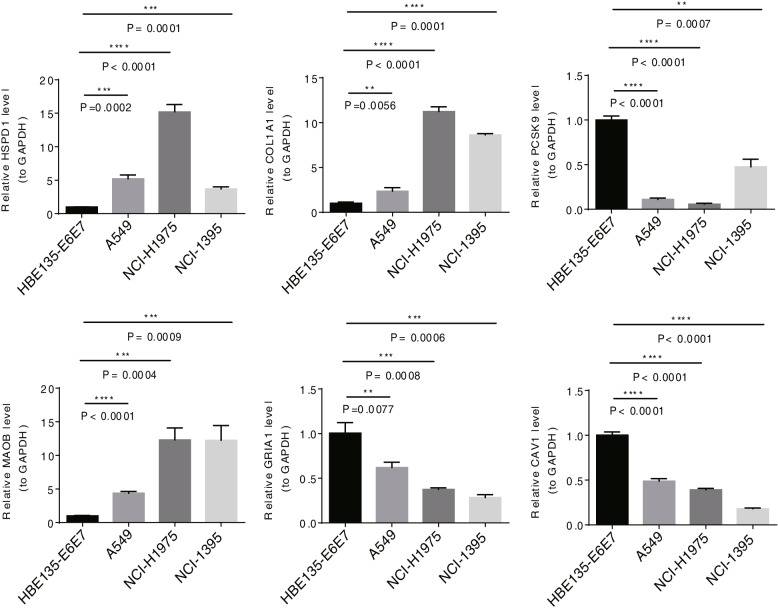
The expression of HSPD1, COL1A1, PCSK9, MAOB, GRIA1, and CAV1 genes in normal cell HBE135-E6E7 and A549, NCI-H1975, and NCI-1395 cell lines

To further validate the biomarker expression, we used qRT-PCR to compare the expression levels of HSPD1, COL1A1, PCSK9, MAOB, GRIA1, and CAV1 genes in normal cell HBE135-E6E7 and A549, NCI-H1975, and NCI-1395 cell lines. qRT-PCR results showed that compared with normal cell lines, the expression of HSPD1, COL1A1, and MAOB genes in A549, NCI-H1975, and NCI-1395 cell lines were significantly upregulated in patients, and the expression of PCSK9, GRIA1, and CAV1 genes in A549, NCI-H1975, and NCI-1395 cell lines were significantly downregulated in patients (Fig. 12).

## Discussion

LUAD in early stage is characterized by no obvious symptoms, and most patients are already in advanced stages when they seek treatment in clinical practice. Although the development of immunotherapy and targeted therapy in recent years has offered a glimpse of long-term survival for patients with advanced LUAD [27, 28], the 5-year survival rate of most advanced LUAD patients is still poor, with 631,000 lung cancer related deaths in 2015 [29]. ERS is a potential target to enhance the efficacy of anti-tumor therapy, especially for immunotherapy. Prognostic models of tumors with endoplasmic reticulum-associated genes have been constructed in certain tumors, such as gliomas. However, prognostic models based on ERS-related genes are rarely reported. The function of ERS in LUAD remains unclear.

In this study, six prognosis-related genes, HSPD1, PCSK9, GRIA1, MAOB, COL1A1, and CAV1 were used to build prognostic model. HSPD1 is a member of the heat shock protein family, and high expression of HSPD1 was related to the poor prognosis of NSCLC patients [30]. PCSK9 belongs to the proprotein convertase family and participate in cholesterol metabolism. PCSK9 inhibition could induce the cell apoptosis of many malignant tumor cells and suppress the progression of lung LUAD [31]. MAOB was related to cancer immune infiltration and linked to the prognosis of LUAD [32]. COL1A1 is an extracellular matrix protein which is highly expressed in LUAD and related to poor prognosis [33], but there were also results showing that COL1A1 had no effect on the OS of female patients with LUAD [34]. CAV1 is a ferroptosis marker and has a regulatory effect on epithelial-mesenchymal transition (EMT) in LUAD. These studies confirmed that these genes were closely associated with the development of LUAD, which provided evidence for this study. In addition, GRIA1 is an ionotropic receptor for glutamate signaling, and has been proved to promote the growth of glioma tumor cell [35]. In this study, GRIA1 was first found to be associated with LUAD and may be a new target. We need more studies to focus on the relevant functions of these ERS-related genes in LUAD.

Enrichment analysis showed that DEGs in the low- and high-risk groups were mainly enriched in processes related to surfactant metabolism, the extracellular matrix (ECM), and collagen. Surfactant metabolism was related to tumor-infiltrating lymphocytes in brain metastasis in LUAD patients, and displayed an inflammatory microenvironment [36]. Surfactant proteins could react with immune cells and suppress the progress of LUAD [37, 38]. ECM transformation in the tumor leads to misexpression of collagen, proteases and integrins in the tumor microenvironment, which promotes tumor progression [39, 40]. Collagen, a major component of extracellular matrix, may also influence tumor initiation and progression. Collagen deposition could increase the incidence of gastric cancer, and collagen rich microenvironment which includes dense fiber could promote tumor progression [41, 42]. Thus, ERSGs may regulate the collagen protofibrils and ECM and it may be involved in the regulation of the microenvironment of LUAD.

TMB is the number of somatic mutations in the tumor cell. The higher the TMB, the higher the level of neoantigen the tumor cells have [43]. In this study, TMB and neoantigen were significantly higher in the high-risk group than in the low-risk group, suggesting that high TMB in the high-risk group may stimulate the proliferation of anti-tumor T cells and anti-tumor response by helping the immune system to recognize LUAD tumor cells. Similarly, we also found TIDE score and PD-L1 expression had remarkable difference in high-risk and how-risk groups. PD-L1 overexpression has been proved to facilitate cancer cells to evade immune surveillance and cause invasion and migration, and is widely used as a predictor of immunotherapy efficacy in LUAD [44, 45]. TIDE score was used to evaluate the immune checkpoint blockade response and cancer immune situation [46]. The TIDE score was significantly higher in the high-risk group in this study, suggesting more severe tumor immune dysfunction and exclusion. The results of TMB、PD-L1 and TIDE score indicated that the high-risk group were more likely to display an immunosuppressed tumor microenvironment. The possible mechanisms were ERS can affect the immune microenvironment of tumors, which can reduce antigen presentation and expression and T cell proliferation [47]. It is reported that the administration of the ERS agent thapsigargin to tumor-rich mice could promote tumor progression and stimulate the accumulation of myeloid-derived suppressor cells (MDSCs) and immunosuppression [48]. All of these suggested that ERS may play an important role in immune suppression of LUAD and ERSGs may be potential novel biomarkers for predicting efficacy of immunotherapies.

Based on public databases,, our study obtained six prognostic genes, (HSPD1, COL1A1, PCSK9, MAOB, GRIA1, and CAV1) and constructed a prognostic model. Additionally, the relevance of prognostic model to clinical traits, vascular-related genes, TMB, immunotherapy response, and drug sensitivity were also explored. This provides a new perspective for the study of ERSGs in LUAD, which has important implications for the diagnosis and treatment of LUAD. However, this study also has a few limitations. Firstly, our analyses were implemented based on public databases, and the results need to be supported and validated by more clinical samples and data. Secondly, the prognostic genes obtained by bioinformatics methods and their mechanisms of action need to be further investigated and validated. Finally, the application of drugs with significantly different sensitivity between high and low risk groups needs further clinical trials and data support. In conclusion, a novel ERS-related risk model was established and validated, which provided a theoretical foundation for ERS-related fields in the study and treatment of LUAD in this study.

## Supplementary Information

Additional file 1: Supplemental Table 1. Primer sequences of qRT-PCR. Supplemental Table 2. The univariate Cox analysis of screening prognostic factors. Supplemental Table 3. The multivariate Cox analysis of screening prognostic factors. Supplemental Table 4. The correlation of risk score and clinical characteristics in training set. Supplemental Table 5. The univariate Cox analysis of independent prognostic analysis in validation set. Supplemental Table 6. The multivariate Cox analysis of independent prognostic analysis in validation set.

## Data Availability

The TCGA-LUAD dataset and GSE31210 analysed during the current study are available in the The Cancer Genome Atlas (TCGA, https://portal.gdc.cancer.gov/) and Gene Expression Omnibus (GEO, https://www.ncbi.nlm.nih.gov/gds) databases, respectively.
