# Supplementary material for: Construction of an endoplasmic reticulum stress-related signature in lung adenocarcinoma by comprehensive bioinformatics analysis
Source: BMC Pulm Med. 2023 May 15;23:172. doi: 10.1186/s12890-023-02443-2 (PMC10186720; doi:10.1186/s12890-023-02443-2)
Supplement: Supplementary file 1 — Additional file 1: Supplemental Table 1. Primer sequences of qRT-PCR. Supplemental Table 2. The univariate Cox analysis of screening prognostic factors. Supplemental Table 3. The multivariate Cox analysis of screening prognostic factors. Supplemental Table 4. The correlation of risk score and clinical characteristics in training set. Supplemental Table 5. The univariate Cox analysis of independent prognostic analysis in validation set. Supplemental Table 6. The multivariate Cox analysis of independent prognostic analysis in validation set. [file 12890_2023_2443_MOESM1_ESM.docx]

Supplemental Table 1. Primer sequences of qRT-PCR

| Sequence name | Primer sequences |
| --- | --- |
| GAPDH F | CCCATCACCATCTTCCAGG |
| GAPDH R | CATCACGCCACAGTTTCCC |
| COL1A1 F | CTCCTGGTCCCCCTGGTC |
| COL1A1 R | GGTTCTTGCGGCTTCCCT |
| PCSK9 F | ACGATGCCTGCCTCTACT |
| PCSK9 R | CAATGATGTCCTCCCCTG |
| MAOB F | GGACAACATGACAATGAAGGA |
| MAOB R | TGACTGAACCCAAAGGCACAC |
| GRIA1 F | TGCCATCTTTGGGTTTTAT |
| GRIA1 R | TCACCTGCCAGTTCTTCTC |
| CAV1 F | GCAGAACCAGAAGGGACAC |
| CAV1 R | CAGACAGCAAGCGGTAAAA |
| HSPD1 F | GTTACCCACAGTCTTTCGCC |
| HSPD1 R | CACACCTCTCCTGATTTCCA |

Supplemental Table 2. The univariate Cox analysis of screening prognostic factors.

| **ID** | **z** | **HR** | **HR.95L** | **HR.95H** | **P value** |
| --- | --- | --- | --- | --- | --- |
| **GAPDH** | 3.575 | 1.474 | 1.192 | 1.824 | 0.000 |
| **SLC2A1** | 3.135 | 1.212 | 1.075 | 1.367 | 0.002 |
| **CAT** | -2.890 | 0.714 | 0.568 | 0.897 | 0.004 |
| **CDK1** | 2.769 | 1.251 | 1.068 | 1.465 | 0.006 |
| **CHEK1** | 2.692 | 1.348 | 1.085 | 1.675 | 0.007 |
| **ADRB2** | -2.681 | 0.708 | 0.551 | 0.911 | 0.007 |
| **HSPD1** | 2.673 | 1.451 | 1.104 | 1.905 | 0.008 |
| **PCSK9** | 2.563 | 1.197 | 1.043 | 1.373 | 0.010 |
| **CFTR** | -2.562 | 0.801 | 0.675 | 0.949 | 0.010 |
| **GRIA1** | -2.545 | 0.401 | 0.198 | 0.811 | 0.011 |
| **MAOB** | -2.489 | 0.810 | 0.686 | 0.956 | 0.013 |
| **SCN4B** | -2.471 | 0.679 | 0.500 | 0.923 | 0.013 |
| **COL1A1** | 2.465 | 1.138 | 1.027 | 1.262 | 0.014 |
| **PABPC1** | 2.356 | 1.353 | 1.052 | 1.739 | 0.018 |
| **CX3CR1** | -2.327 | 0.757 | 0.599 | 0.957 | 0.020 |
| **CAV1** | 2.295 | 1.161 | 1.022 | 1.319 | 0.022 |
| **RAD51** | 2.285 | 1.303 | 1.038 | 1.636 | 0.022 |
| **MAOA** | -2.131 | 0.873 | 0.770 | 0.989 | 0.033 |

Supplemental Table 3. The multivariate Cox analysis of screening prognostic factors.

| **ID** | **coef** | **HR** | **HR.95L** | **HR.95H** | **P value** |
| --- | --- | --- | --- | --- | --- |
| **HSPD1** | 0.321 | 1.378 | 1.041 | 1.823 | 0.025 |
| **PCSK9** | 0.233 | 1.263 | 1.101 | 1.448 | 0.001 |
| **GRIA1** | -0.822 | 0.439 | 0.197 | 0.979 | 0.044 |
| **MAOB** | -0.159 | 0.853 | 0.703 | 1.034 | 0.105 |
| **COL1A1** | 0.124 | 1.132 | 1.011 | 1.268 | 0.032 |
| **CAV1** | 0.251 | 1.286 | 1.127 | 1.467 | 0.000 |

Supplemental Table 4. The correlation of risk score and clinical characteristics in training set.

|  | **Total** | | **high** | **low** | ***P* value** |
| --- | --- | --- | --- | --- | --- |
|  | **(N=226)** | | **(N=119)** | **(N=107)** |  |
| **Gender** |  | |  |  |  |
| Female | 123 (54.4%) | | 57 (47.9%) | 66 (61.7%) | 0.052 |
| Male | 103 (45.6%) | | 62 (52.1%) | 41 (38.3%) |  |
| **Age (years)** | |  |  |  |  |
| >=60 | 163 (72.1%) | | 80 (67.2%) | 83 (77.6%) | 0.113 |
| <60 | 63 (27.9%) | | 39 (32.8%) | 24 (22.4%) |  |
| **M Stage** |  | |  |  |  |
| M0 | 208 (92.0%) | | 106 (89.1%) | 102 (95.3%) | 0.137 |
| M1 | 18 (8.0%) | | 13 (10.9%) | 5 (4.7%) |  |
| **N Stage** |  | |  |  |  |
| N0 | 143 (63.3%) | | 57 (47.9%) | 86 (80.4%) | <0.001 |
| N1 | 49 (21.7%) | | 35 (29.4%) | 14 (13.1%) |  |
| N2 | 33 (14.6%) | | 26 (21.8%) | 7 (6.5%) |  |
| N3 | 1 (0.4%) | | 1 (0.8%) | 0 (0%) |  |
| **T Stage** |  | |  |  |  |
| T1 | 69 (30.5%) | | 25 (21.0%) | 44 (41.1%) | 0.0053 |
| T2 | 127 (56.2%) | | 73 (61.3%) | 54 (50.5%) |  |
| T3 | 17 (7.5%) | | 11 (9.2%) | 6 (5.6%) |  |
| T4 | 13 (5.8%) | | 10 (8.4%) | 3 (2.8%) |  |
| **Stage** |  | |  |  |  |
| Stage I | 114 (50.4%) | | 40 (33.6%) | 74 (69.2%) | <0.001 |
| Stage II | 54 (23.9%) | | 35 (29.4%) | 19 (17.8%) |  |
| Stage III | 40 (17.7%) | | 31 (26.1%) | 9 (8.4%) |  |
| Stage IV | 18 (8.0%) | | 13 (10.9%) | 5 (4.7%) |  |

Supplemental Table 5. The univariate Cox analysis of independent prognostic analysis in validation set.

| **Variable** | **Coef** | **HR** | **HR.95L** | **HR.95H** | ***P* value** |
| --- | --- | --- | --- | --- | --- |
| **Age** | 0.009 | 1.009 | 0.988 | 1.031 | 0.383 |
| **Gender** | (0.049) | 0.952 | 0.639 | 1.417 | 0.808 |
| **M** | 0.742 | 2.101 | 1.081 | 4.083 | 0.029 |
| **N** | 0.595 | 1.813 | 1.457 | 2.257 | 0.000 |
| **Stage** | 0.523 | 1.687 | 1.397 | 2.036 | 0.000 |
| **T** | 0.382 | 1.465 | 1.169 | 1.837 | 0.001 |
| **Risk score** | 0.200 | 1.221 | 1.127 | 1.323 | 0.000 |

Supplemental Table 6. The multivariate Cox analysis of independent prognostic analysis in validation set.

| **ID** | **coef** | **HR** | **HR.95L** | **HR.95H** | ***P* value** |
| --- | --- | --- | --- | --- | --- |
| **N** | 0.251 | 1.286 | 0.945 | 1.750 | 0.110 |
| **STAGE** | 0.357 | 1.429 | 1.091 | 1.870 | 0.010 |
| **Risk score** | 0.146 | 1.157 | 1.063 | 1.260 | 0.001 |
